# Bradycardia, Renal Failure, Atrioventricular Nodal Blockade, Shock, and Hyperkalemia (BRASH) Syndrome Associated With Baclofen and Diazepam

**DOI:** 10.7759/cureus.97799

**Published:** 2025-11-25

**Authors:** Yasutoshi Yamamoto, Akihiro Matsushita, Tomoyo Yamashita, Katsuji Tanaka

**Affiliations:** 1 Department of Pediatrics, Nishinomiya Sunago Medical and Welfare Center, Nishinomiya, JPN; 2 Department of Pediatrics, Graduate School of Medicine, Osaka Metropolitan University, Osaka, JPN

**Keywords:** atrioventricular nodal-blocking agents, bradycardia, brash syndrome, hyperkalemia, impaired renal function

## Abstract

Bradycardia, renal failure, atrioventricular nodal blockade (AVNB), shock, and hyperkalemia (BRASH) syndrome is a relatively recently recognized clinical entity that can result in life-threatening complications. Although AVNB caused by beta blockers and calcium channel blockers is central to disease onset, baclofen and diazepam have not previously been implicated. We report a case of BRASH syndrome triggered by the combination of baclofen and diazepam, without involvement of other known AVNB agents.

A 60-year-old male with cerebral palsy and chronic kidney disease was found unresponsive. He had started minocycline for a urinary tract infection five days prior and had not consumed food on the day of presentation. Initial vital signs revealed bradycardia, hypotension, and hypothermia. Laboratory testing showed mild hyperkalemia. Bradycardia and hypotension improved gradually with fluid resuscitation. Based on the constellation of findings, including bradycardia, renal failure, hypotension, and hyperkalemia, the patient was retrospectively diagnosed with BRASH syndrome associated with baclofen and diazepam, the only medications he was taking with AVNB properties. Although his renal function deteriorated and hyperkalemia progressed 15 days after onset, appropriate dietary management and fluid intake successfully prevented recurrence.

This case demonstrates that the combination of baclofen and diazepam can induce BRASH syndrome. Maintaining adequate hydration proved effective in preventing recurrence, despite worsening renal function and increasing hyperkalemia. In patients with limited renal reserve who present with bradycardia and hyperkalemia while receiving baclofen and diazepam, BRASH syndrome should be considered.

## Introduction

BRASH syndrome, an acronym for bradycardia, renal failure, atrioventricular nodal blockade (AVNB), shock, and hyperkalemia, is a recently identified clinical entity that was first described in 2016 by Farkas [1]. It represents a vicious cycle in which renal failure causes hyperkalemia and the accumulation of AVNB agents, leading to bradycardia and hypoperfusion, which in turn worsen renal failure [1]. The pathophysiological hallmark of BRASH syndrome is the synergistic effect of hyperkalemia and AVNB agents, which together contribute to the development of bradycardia. This synergistic interplay between hyperkalemia and AVNB agents can result in rapid clinical deterioration if not promptly identified and managed [2]. Conventional Advanced Cardiovascular Life Support (ACLS) protocols may be ineffective in BRASH syndrome. However, once identified, treatment is straightforward and recovery is often rapid. Despite increasing recognition, BRASH syndrome remains underdiagnosed - frequently mistaken for isolated hyperkalemia, with the contribution of AVNB agents overlooked. In BRASH syndrome, even mild hyperkalemia can induce bradycardia through synergy with AVNB agents, whereas hyperkalemia alone typically causes bradycardia only at higher potassium levels. Bradycardia without classic electrocardiographic (ECG) findings (for example, QRS widening or peaked T waves) should prompt consideration of BRASH syndrome. Unlike AVNB agents overdose, which often involves large ingestions and may or may not include hyperkalemia, BRASH syndrome typically occurs in patients taking prescribed doses, with persistently elevated potassium levels. The syndrome is most commonly associated with AVNB agents such as beta blockers and calcium channel blockers [1]. These medications, especially in patients with underlying renal impairment, can precipitate or amplify the cycle of BRASH. However, other AVNB agents, including potassium channel blockers, sodium channel blockers, and digoxin, may also contribute, although they are less frequently reported [3-8].

Baclofen, a gamma-aminobutyric acid type B (GABA-B) receptor agonist used for spasticity, and diazepam, a benzodiazepine, are both known to cause central nervous system depression and hypotension [9]. Additionally, baclofen has been shown to activate GABA-B receptors in cardiac myocytes, mirroring its neuronal effects [10]. Through G-protein signaling, it may inhibit voltage-dependent calcium channels such as Cav2.1 and Cav2.3, thereby reducing calcium influx, delaying the upstroke of the action potential in the atrioventricular (AV) node, and potentially suppressing AV nodal conduction [11]. Recent findings also suggest that AV nodal pacemaker cells (AVNPCs) possess a complete GABAergic system, including GABA receptors, transporters, and metabolic enzymes, implicating GABA in the modulation of cardiac electrical activity and the development of AV block [12]. Similarly, benzodiazepines, while primarily acting on central GABA type A (GABA-A) receptors, have been reported to interact with peripheral BZ-3 receptors in cardiac tissue. Benzodiazepine agonists may reduce T-type and L-type calcium currents, functioning as weak calcium channel blockers and potentially contributing to AV block in overdose scenarios [13]. Furthermore, diazepam has been shown to exert a direct negative dromotropic effect on the AV node, independent of autonomic influences [14]. While their direct role in AVNB is not well established, their combined sedative and hypotensive effects may potentiate bradycardia and renal hypoperfusion, especially in vulnerable populations [9,15]. To our knowledge, the combination of baclofen and diazepam has not previously been reported as a causative factor of BRASH syndrome.

This report presents a case of BRASH syndrome induced by baclofen and diazepam in the absence of other AVNB agents. This case highlights the need for heightened clinical awareness of atypical pharmacologic contributors to BRASH syndrome, particularly in patients with limited renal reserve.

## Case presentation

A 60-year-old male with severe motor and intellectual disabilities secondary to cerebral palsy due to perinatal asphyxia was found unresponsive at a residential hospital.

Due to persistent muscle hypertonia unresponsive to botulinum toxin injections, oral diazepam (2.5 mg daily at 21:00) was initiated three years before illness onset. Two years prior, oral baclofen (30 mg daily, divided into three doses at 6:00, 12:00, and 18:00 h) was added due to worsening muscle tension. He had a history of recurrent urinary tract infections (UTIs) related to urinary stones and a duplicated ureter and renal pelvis on the right side. He developed stage 3b chronic kidney disease (CKD) following multiple courses of antibiotics for infections caused by extended-spectrum beta-lactamase-producing *Escherichia coli*. His most recent estimated glomerular filtration rate based on serum creatinine (eGFRcre) was 41 mL/min/1.73 m², consistent with CKD stage 3b. One month before the presentation, he had lost 4.3 kg over a three-month period (from 43.2 kg to 38.9 kg). Five days before symptom onset, he began taking oral minocycline (200 mg daily) for a UTI. The day before onset, he experienced transient generalized weakness, neck instability during position changes, and impaired pursuit eye movements, although his eyes remained open and blood pressure (BP) remained stable (117/77 mmHg at 7:00 PM). He returned to baseline hypertonia by 8:00 PM. He did not consume food on the day of onset.

Initial vital signs revealed bradycardia (31 beats per minute; bpm), hypotension (82/58 mmHg), and hypothermia (33.3°C) (Table 1). Oxygen saturation was 100% on pulse oximetry. Telemetry confirmed sinus bradycardia at 31 bpm, without disappearance of P waves, QRS widening, or a sine-wave pattern (Table 1). On physical examination, the patient grimaced in response to painful stimuli, although reactions were diminished. His medications included baclofen (30 mg daily), diazepam (2.5 mg daily), famotidine (20 mg daily), Macrogol 4000 plus electrolytes (Movicol, EA Pharma, Tokyo, Japan), and minocycline (200 mg daily).

**Table 1:** Changes in vital signs from illness onset Marked bradycardia and hypotension were observed on Day 1. No episodes of bradycardia below 40 bpm or significant hypotension were noted on Days 16, 19, or 24. N/A: not available.

Vitals	Day 1	Day 16	Day 19	Day 24
Body Temperature (℃)	33.3	34.9	33.6	35.7
Pulse (bpm)	31	50	48	50
Blood Pressure (mmHg)	82/58	107/58	120/71	N/A

Laboratory test results showed normochromic, normocytic anemia (hemoglobin (Hb) 8.1 g/dL; hematocrit 23.5%). Biochemistry results revealed a glucose level of 108 mg/dL; blood urea nitrogen (BUN), 38.1 mg/dL; creatinine (Cre), 1.27 mg/dL; potassium (K), 5.4 mEq/L; sodium (Na), 138 mEq/L; thyroid-stimulating hormone (TSH), 1.54 mIU/L; and free T4, 1.06 ng/dL (Table 2).

**Table 2:** Changes in laboratory findings from illness onset Mild hyperkalemia was observed on Day 1. Hyponatremia and hyperkalemia noted on Day 16 improved by Day 24 following administration of loop diuretics. BUN: blood urea nitrogen; Cre: creatinine; eGFRcre: estimated glomerular filtration rate based on serum creatinine; eGFRcys: estimated glomerular filtration rate based on serum cystatin C; K: potassium; Na: sodium; TP: total protein; Alb: albumin; TSH: thyroid-stimulating hormone; WBC: white blood cell; Hb: hemoglobin; Ht: hematocrit; CRP: C-reactive protein; N/A: not available.

Labs	Day 1	Day 16	Day 19	Day 24	Reference
BUN (mg/dL)	38.1	26.7	24.4	23.2	8.0-22.0
Cre (mg/dL)	1.27	1.78	1.53	1.18	0.61-1.04
Cystatin C (mg/L)	N/A	N/A	N/A	2.24	0.63-0.95
eGFRcre (mL/min/1.73 m^2^)	46.12	31.88	37.62	49.98	>60
eGFRcys (mL/min/1.73 m^2^)	N/A	N/A	N/A	27.95	>60
K (mEq/L)	5.4	6.1	5.6	4.7	3.5-5.3
Na (mEq/L)	138	122	130	134	135-150
TP (g/dL)	6	5.8	5.9	5.7	6.5-8.3
Alb (g/dL)	2.1	2.3	2.3	2.3	3.8-5.3
TSH (mIU/L)	1.54	N/A	N/A	N/A	0.54-4.54
Free T4 (ng/dL)	1.06	N/A	N/A	N/A	0.97-1.72
Glucose (mg/dL)	108	N/A	N/A	N/A	70-100
WBC (/mL)	3000	4600	3200	4800	3900-9800
Hb (g/dL)	8.1	6.9	7.1	6.5	13.5-17.6
Ht (%)	23.5	19.8	20.8	19.8	39.8-51.8
CRP (mg/dL)	1.19	0.25	0.4	1.5	<0.30
Erythropoietin (mIU/mL)	N/A	8.6	N/A	N/A	4.2-23.7

To manage hypotension, unresponsiveness, and dehydration (suggested by elevated BUN, UTI, and poor intake), acetated Ringer’s solution was administered. After 3 h, the patient regained consciousness and was able to eat. Vital signs improved: bradycardia (39 bpm), BP (115/57 mmHg), and temperature (33.9°C). After 18 h, further improvements were noted: heart rate 53 bpm, blood pressure 112/60 mmHg, and temperature 35.5°C.

On days 16 and 19 after illness onset, following a 10-day course of trimethoprim/sulfamethoxazole for UTI, the patient developed hyponatremia and hyperkalemia (Na/K: 122 mEq/L/6.1 mEq/L on Day 16; 130 mEq/L/5.6 mEq/L on Day 19; Table 2) without worsening hypotension or bradycardia (Table 1). He maintained adequate oral intake and did not require further fluid therapy. Electrolyte abnormalities resolved after eight days of oral loop diuretics (Na/K: 134 mEq/L/4.7 mEq/L on Day 24; Table 2). Renal anemia was confirmed on Day 16 (Hb 6.9 g/dL; erythropoietin 8.6 mIU/mL; Table 2) and was treated with subcutaneous epoetin beta starting on Day 24. Hb levels improved to 11.2 g/dL at four months post-treatment.

**Figure 1:**
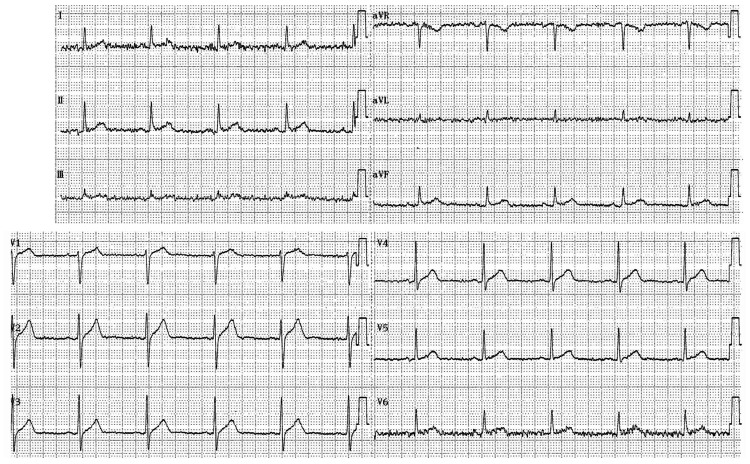
ECG findings before administration of baclofen and diazepam ECG was recorded five years prior to the onset of BRASH syndrome, two years before the initiation of diazepam, and three years before the initiation of baclofen. Sinus rhythm at 59 bpm was observed. No QT prolongation (QTcF 398 ms) or PR interval prolongation (PR 164 ms) was detected. ECG: electrocardiographic; BRASH: bradycardia, renal failure, atrioventricular nodal blockade, shock, and hyperkalemia; QTcF: corrected QT interval using Fridericia’s formula.

**Figure 2:**
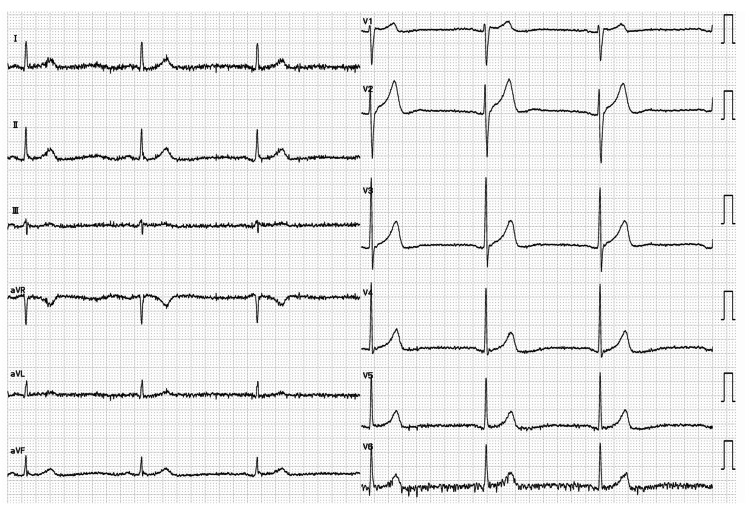
ECG findings during administration of baclofen and diazepam ECG was recorded on Day 68, 17 days after initiating baclofen dose reduction from 30 mg/day to 20 mg/day. Sinus bradycardia (36 bpm), mild QT prolongation (QTcF 433 ms), and mild PR interval prolongation (184 ms) were observed. The diazepam dose remained unchanged at 2.5 mg. ECG: electrocardiographic; QTcF: corrected QT interval using Fridericia’s formula.

**Figure 3:**
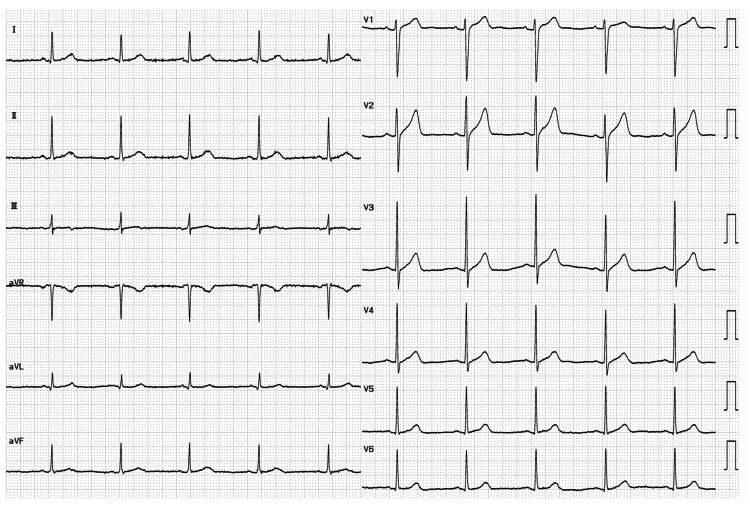
ECG findings after discontinuation of baclofen and diazepam ECG was recorded one year and nine months after the onset of BRASH syndrome, one month after discontinuation of diazepam, and five months after discontinuation of baclofen. Sinus rhythm at 61 bpm was observed. No QT prolongation (QTcF 396 ms) or PR interval prolongation (PR 144 ms) was detected. ECG: electrocardiographic; BRASH: bradycardia, renal failure, atrioventricular nodal blockade, shock, and hyperkalemia; QTcF: corrected QT interval using Fridericia’s formula.

Based on the presence of bradycardia, renal failure, hypotension, and hyperkalemia, the patient was retrospectively diagnosed with BRASH syndrome. To avoid withdrawal symptoms, baclofen tapering began a month after illness onset and continued over a year until discontinuation at 15 months. On Day 68 (17 days after initiating baclofen dose reduction from 30 mg/day to 20 mg/day), elevated BP (139/64 mmHg) and transient sinus bradycardia were observed immediately following the third administration of epoetin beta. These findings were accompanied by mild prolongation of PR and QT intervals, slightly greater than those recorded in the absence of baclofen and diazepam (see Table 3; Figures 1, 2). Renal function remained stable (serum creatinine 1.18 mg/dL), and no hyperkalemia was detected (serum potassium 4.4 mEq/L). Without pharmacological intervention, both BP and bradycardia resolved spontaneously within one day under clinical observation. Diazepam was subsequently discontinued 18 months after onset. The withdrawal period was uneventful. Throughout, the patient’s pulse and BP remained stable (40-70 bpm; 100-110/50-60 mmHg; see Figure 3). Since the initiation of appropriate nutritional and fluid support following the onset of BRASH syndrome, no recurrence has been observed over a two-year and four-month period. This includes the 13 months following baclofen discontinuation, of which nine months were after both baclofen and diazepam had been stopped, further supporting the preventive effect of drug withdrawal and supportive care.

**Table 3:** Electrocardiographic findings with or without baclofen and diazepam Mild prolongation of PR and QT intervals was observed during administration of baclofen (20 mg) and diazepam (2.5 mg). No prolongation of PR or QT intervals was noted prior to initiation or after discontinuation of both medications. QTcF: corrected QT interval using Fridericia’s formula.

Treatment phase	Baclofen	Diazepam	PR (ms)	QTcF (ms)
Pre-treatment	(-)	(-)	164	398
During treatment	(+)	(+)	184	433
Post-withdrawal	(-)	(-)	144	396

## Discussion

Two key clinical points were identified: (1) a combination of baclofen and diazepam can cause BRASH syndrome; and (2) dehydration avoidance was effective in preventing recurrence of the syndrome, despite further deterioration of renal function and progression of hyperkalemia.

First, a combination of baclofen and diazepam can induce BRASH syndrome [1]. AVNB is essential for the development of BRASH syndrome. The most commonly used AVNB-inducing medications are beta blockers and calcium channel blockers [1]. Prior case reviews have also identified potassium channel blockers, sodium channel blockers, and digoxin as AVNB agents contributing to BRASH syndrome [3-8] (Table 4). Unlike typical cases reported in older patients (mostly over 70 years old) requiring interventions such as calcium supplementation, insulin with dextrose, or catecholamines, our case featured a relatively younger patient (60 years old) who recovered with fluid therapy alone. This suggests a milder presentation and highlights the potential for conservative management in select cases. Although baclofen is a muscle relaxant, it has been reported to exert AVNB effects, although the underlying mechanisms remain unclear [16,17]. Diazepam, a benzodiazepine used as an anxiolytic and muscle relaxant, also exhibits AVNB properties, possibly due to weak calcium channel blockade [13,14,18]. Based on clinical findings, including bradycardia, renal failure, hypotension, and hyperkalemia, our patient was diagnosed with BRASH syndrome. Baclofen and diazepam, the only medications with AVNB effects in this case, likely contributed to the bradycardia and subsequent development of BRASH syndrome. Notably, BRASH syndrome often occurs at therapeutic, not toxic, doses of AVNB agents, distinguishing it from bradycardia or shock caused by pure AVNB effects [1]. In this case, bradycardia and shock emerged with a normal dose of baclofen (30 mg daily) and a low dose of diazepam (2.5 mg daily; typical therapeutic range is 4-20 mg daily), not toxic doses. Additionally, BRASH syndrome may develop in the presence of only mild hyperkalemia, particularly when patients are using AVNB agents and ECG changes are limited. This further distinguishes BRASH-related bradycardia from that caused by pure hyperkalemia [1]. Our patient experienced bradycardia associated with mild hyperkalemia (absolute value K 5.4 mEq/L) while taking baclofen and diazepam, both known to possess AVNB effects (Table 2). ECG showed only sinus bradycardia without other significant changes. These findings also support the diagnosis of BRASH syndrome over pure hyperkalemia. To our knowledge, this is the first reported case of BRASH syndrome associated with the combination of baclofen and diazepam.

**Table 4:** Atrioventricular nodal blockers other than calcium channel blockers and beta blockers associated with BRASH syndrome F: female; M: male; K^+^: potassium ion; Na^+^: sodium ion; Ca^2+^: calcium ion; BRASH: bradycardia, renal failure, atrioventricular nodal blockade, shock, and hyperkalemia.

Authors	Age/sex	Drugs	Class	Pharmacological action
Juvet et al., 2013 [8]	85/F	Sotarol	III	K^+^ channel blocker
Zaidi et al., 2019 [7]	88/F	Ranolazine	I	Late Na^+^ current inhibitor
Pata et al., 2022 [6]	58/M	Sotarol	III	K^+^ channel blocker
Phuyal et al., 2023 [5]	97/F	Amiodarone	III	Multi-channel blocker (K^+^, Na^+^, Ca^2+^)
Shah et al., 2023 [4]	81/M	Digoxin	II	Muscarinic M_2 _receptor activator
Saeed et al., 2023 [3]	74/F	Digoxin	II	Muscarinic M_2 _receptor activator
Our case	60/M	Baclofen, diazepam	Unknown, Unknown	Unknown, Possibly weak Ca^2+^ channel blocker

Second, dehydration avoidance was effective in preventing recurrence of the syndrome, despite further deterioration of renal function and worsening hyperkalemia. Dehydration, gastroenteritis, and inadequate dietary intake are recognized as important triggers or underlying conditions for the development of BRASH syndrome [1]. On Day 16, 15 days after the initial presentation, the patient exhibited worsened renal function (Cre 1.78 mg/dL vs. 1.27 mg/dL on Day 1; Table 2) and increased hyperkalemia (K 6.1 mEq/L vs. 5.4 mEq/L on Day 1; Table 2), likely related to antibiotic treatment for UTI. However, the patient maintained adequate dietary intake and did not experience recurrence of bradycardia or hypotension (pulse 50 bpm, blood pressure 107/58 mmHg on Day 16; Table 1). These findings suggest that sufficient diet and fluid intake may help prevent recurrence of BRASH syndrome associated with baclofen and diazepam.

Non-cardiovascular medications such as baclofen and benzodiazepines, including lorazepam and diazepam, are known not only for their AVNB effects but also for QT prolongation [19,20]. In this case, the patient exhibited both PR and QT prolongation during baclofen and diazepam use, whereas neither PR nor QT prolongation was observed in the absence of these medications (Table 3, Figures 1-3). Although PR or QT prolongation is not commonly caused by either drug alone, their concurrent use may amplify these effects.

## Conclusions

A combination of baclofen and diazepam can induce BRASH syndrome. Dehydration avoidance was effective in preventing recurrence, even in the presence of deteriorated renal function and progressive hyperkalemia. Patients with reduced renal reserve who are taking baclofen and diazepam are at risk of developing BRASH syndrome and should be advised to maintain an adequate diet and hydration, especially during infections. Just as BRASH syndrome may be overlooked in patients with impaired renal function using beta blockers or calcium channel blockers, it can also be missed in patients taking baclofen and diazepam. The mechanisms by which baclofen and diazepam contribute to BRASH syndrome remain unclear. As this is a single-case report, the causal relationship between drug administration and syndrome onset remains speculative. Further accumulation of cases and research, including pharmacologic confirmation, is needed to better understand the pathophysiology of baclofen-diazepam-induced BRASH syndrome.
